# Sustainability in the Aerospace, Naval, and Automotive Supply Chain 4.0: Descriptive Review

**DOI:** 10.3390/ma13245625

**Published:** 2020-12-10

**Authors:** Magdalena Ramirez-Peña, Pedro F. Mayuet, Juan Manuel Vazquez-Martinez, Moises Batista

**Affiliations:** School of Engineering, University of Cádiz, Avenue Universidad de Cádiz, 10, 11519 Puerto Real-Cadiz, Spain; pedro.mayuet@uca.es (P.F.M.); juanmanuel.vazquez@uca.es (J.M.V.-M.)

**Keywords:** sustainability, supply chain management, manufacturing system, automotive, aerospace, shipbuilding, transports

## Abstract

The search for sustainability in the Supply Chain (SC) is one of the tasks that most concerns business leaders in all manufacturing sectors because of the importance that the Supply Chain has as a transversal tool and due to the leading role that it has been playing lately. Of all the manufacturing sectors, this study focuses on the aerospace, shipbuilding, and automotive sectors identified as transport. The present study carries out a descriptive review of existing publications in these three sectors in relation to the sustainability of the Supply Chain in its 4.0 adaptation as an update in matters that are in constant evolution. Among the results obtained, Lean practices are common to the three sectors, as well as different technologies focused on sustainability. Furthermore, the results show that the automotive sector is the one that makes the greatest contribution in this sense through collaborative programs that can be very useful to the other two sectors, thus benefiting from the consequent applicable advantages. Meanwhile, the Aerospace and Shipbuilding sectors do not seem to be working on promoting a sustainable culture in the management of the Supply Chain or on including training programs for their personnel in matters related to Industry 4.0.

## 1. Introduction

It can be said that a Supply Chain is composed of all the interested parties: customers, suppliers, manufacturers, transporters, warehousemen, etc. Each organization includes all the functions involved in it starting from the development of the new product, marketing, manufacturing, finance, to customer service and whose purpose is to satisfy the needs of the customer while generating profits in the process for itself [1].

Each Supply Chain will be divided into different stages, and within each stage, several actors can coexist, so it should really be called a Supply Network. All stages are connected through the flow of products, information, and funds—in both directions—aimed at maximizing the total value generated by the Supply Chain. The success of a Supply Chain should not be measured at each stage but in its total profitability. Therefore, the success of a Supply Chain lies in the efficiency of its management [2].

In addition, Supply Chain must adapt both to changes in technology and to customer requirements in order to remain competitive. The manufacturing Supply Chain is of the pull type as the processes are carried out in response to the request of the customer, which is also known as a reactive process [3]. 

Each connection between the stages of the Supply Chain (supplier–manufacturer–distributor–retailer–customer) has the processes required for each process cycle (sales order cycle, replenishment cycle, manufacturing cycle, procurement cycle), and these connection processes are divided into sub-processes at the same time [4]. The cycle view is useful when establishing information systems to support Supply Chain operations when considering operational decisions because it establishes the roles and responsibilities of each member and the expected outcome of each process.

**Figure 1:**
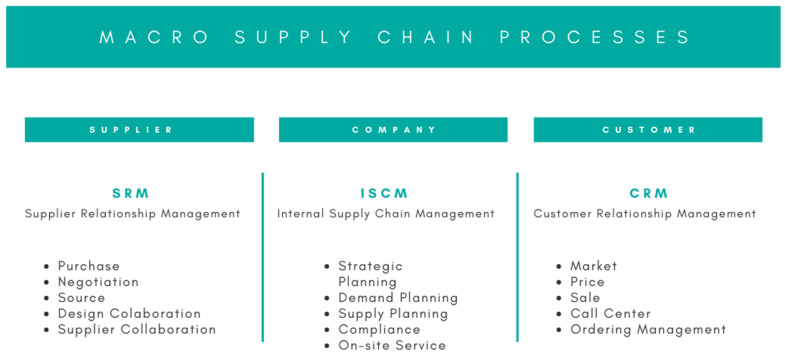
Macro Supply Chain processes adapted from [1].

Therefore, Supply Chain activities are framed within three macro processes: CRM, Customer Relationship Management; ISCM, Internal SCM; and SRM, Supplier Relationship Management. Figure 1 details these three framework processes [1].

Information is disrupted as it moves up the chain because the information shared in the stages is incomplete. The lack of coordination can be called “the whip effect”. This lack of coordination damages relations between the different stages where there is a tendency to blame other stages thinking that theirs is doing well, which causes a loss of trust between the different stages and makes coordination efforts difficult [5].

In the present case, transport companies tend to report on greenhouse gas emissions, fuel consumption, and transport efficiency. From an environmental perspective, they report on four categories: energy consumption, water consumption, greenhouse gas emissions, and waste generation.

The role of sustainability in the Supply Chain today has become crucially important in both its design and the operations that concern it while improving its performance [6]. The framework presented by the United Nations World Summit in 2005 identifies three pillars on which sustainable economic, environmental, and social development rests.

In order to build a more sustainable Supply Chain, companies must clearly define the reasons for developing more sustainable approaches to fuel interest from customers who are reluctant to pay more for sustainable products [7].

Therefore, the aim of this article is to explore the advances that exist in the three manufacturing sectors: Aerospace, Shipbuilding, and Automotive in terms of sustainable Supply Chain management. At the same time, common areas and possible synergies between the three sectors will be identified. 

## 2. Materials and Methods 

**Figure 2:**
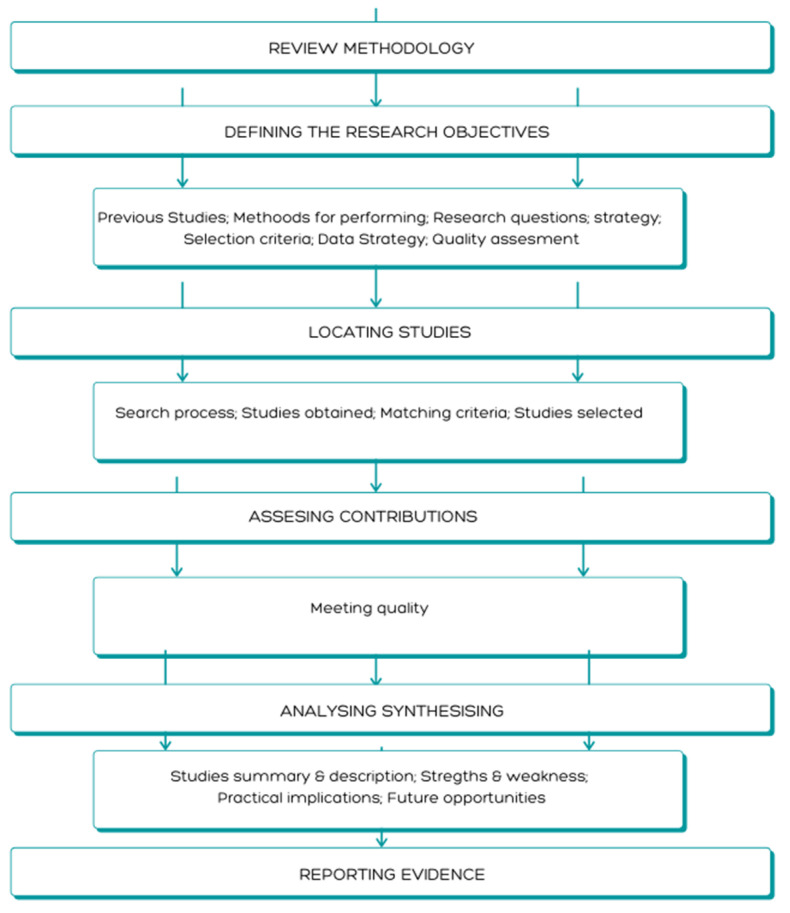
Research methodology for this study.

The methodology carried out in this work is shown in Figure 2. It is a descriptive review in order to provide the existing advances on Supply Chain in the three big sectors that compose the transport manufacturing such as aerospace, naval, and automotive. Many advances are being made in each of these areas individually with respect to sustainability-focused supply chain management, but a descriptive review of the three areas together will provide an update for people working in the same fields in different areas. It is in this sense where it is intended to highlight that synergies are possible. Both aerospace and shipbuilding coincide in the type of production, while the automotive sector is mass production. However, there are related fields that would take advantage of advances in each production system or even sector. Hence, the managers dedicated to these fields can be nourished with the studies published by the scientific community serving as a strategic tool that allows them to update the various aspects addressed in the study. Therefore, the aim is to find out what work is being done in these three areas in terms of the progress of Industry 4.0 (I4.0) together with sustainability [8].

The study will be developed in five stages, starting with the definition of the research objective, mentioned above, until the evidence is reported once it has been analyzed [9].

To carry out the bibliographic search, Scopus was used as the main database in which the main journals and conference papers will be studied, as well as some book chapters and review articles. 

To establish the search strategy, the descriptors “Supply Chain Management”, “4.0”, and each of the three sectors—“aerospace”, “shipbuilding”, “automotive”—were used as arguments. No exclusion criteria were established with respect to time due to the inclusion of the term 4.0 as a descriptor that acts as a limiter.

**Figure 3:**
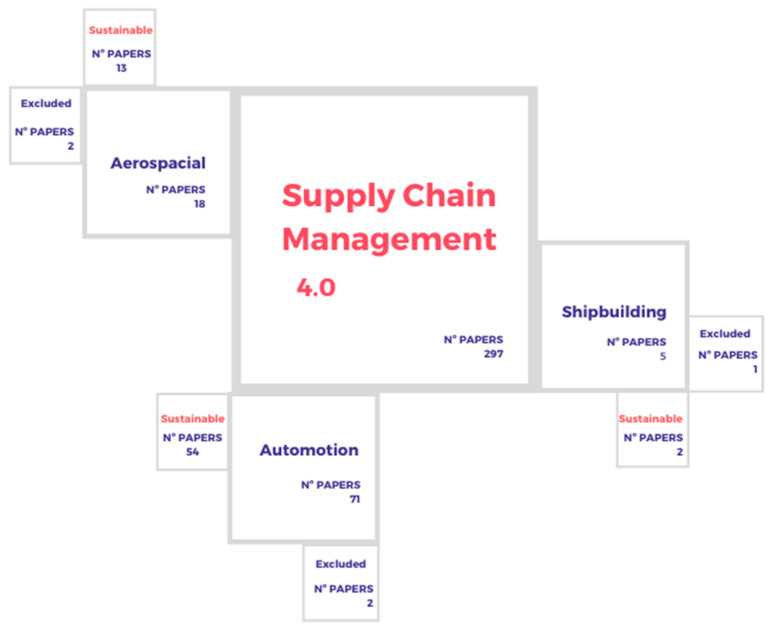
Articles considered from each sector.

A total of 297 articles were found, to which the criteria of scientific quality were applied. Subsequently, duplicates were eliminated, and the abstracts and conclusions were not read until the articles were selected to be read in their entirety. The distribution of publications used in the study is shown in Figure 3.

## 3. Results and Discussion

### 3.1. Aerospace

Starting with the aerospace sector and considering the above-mentioned search arguments, a total of 18 articles are established that deal with this industry, of which 13 include sustainability in their content. Only two articles have been excluded because the criteria established were not met.

After evaluating the adaptation that this type of industry whose production engineer-to-order is characterized by the activities that must be added in order to comply with the established lead time, whether in terms of commercial management, procurement, production, or logistics and distribution in the case of the Supply Chain, the difficulty that smaller companies face in adapting to Industry 4.0 becomes clear. While large companies are more aware of the changes they must make in this adaptation, the Supply Chain is made up of these and other smaller and less developed companies in terms of both resources and organizational capacity for the integration of Industry 4.0, one of their concerns being the susceptibility to external breakdown [10].

Engineers, to order environments, develop Lean methodologies to accelerate delivery time among other techniques. Lean practices such as Just In Time (JIT) and Visual Management show how certain areas improve the potential impacts of business performance as well as the overall Supply Chain [11]. In addition to Lean, Green is another paradigm that focuses on the requirements that I4.0 makes, from product and process design, production planning and control, and communication with suppliers. Furthermore, the flexibility in the development of shared communication with suppliers is a fundamental requirement for the competitiveness of the Supply Chain [12]. To achieve this required competitiveness, in addition to enhancing management and sustainability in the Supply Chain, aerospace companies demonstrate the impact of product life management (PLM) systems by managing the entire product life cycle, from the first marketing idea to the after-sales service [13].

With regard to the digitalization of the framework processes of the Supply Chain, technologies such as the Internet of Things become important for companies in the sector interested in the transformation of Industry 4.0. Management principles that improve performance throughout the company focused on the involvement of employees in decision making, and two applications are the most suitable for the implementation of this technology: TQM (total quality management), which is a CRM (Customer Relationship Management) application that allows centralizing in a single database all interactions between a company and customers and the management of relationships with suppliers, and SRM (Supplier Relationship Management), with the intention of establishing positive relationships with the company. In addition, these are also used in the reduction of carbon emissions and the adoption of Green concepts [14].

It could be said that the Internet of Things collaborates closely on energy management in smart factories, smart logistics and transport, and creating smart business models. This is done in four main areas: (1) designing incentive mechanisms to promote green consumer behavior; (2) improving visibility throughout the product life cycle; (3) increasing system efficiency while reducing development and operational costs; and (4) encouraging sustainability monitoring and reporting performance in Supply Chain networks [15].

Moreover, the Internet of Things becomes more important in terms of the need to be able to visualize information in real time [16], as well as the existing improvement in after-sales services achieved through the sensors placed in its products, together with the Big Data technology, which reports on their performance, defects, and usage patterns in the hands of the customer. This fact has changed the business model, and the manufacturer has become the solution to the problem [17].

In this way, the importance of Big Data is confirmed due to the critical challenge that these factories have to process so much information. These intelligent systems are capable of monitoring and controlling the processes of the Supply Chain as well as providing information on breakdowns for the entire system of planning and control of production and finally providing useful solutions to employees [16].

With respect to Additive Manufacturing, it plays an important role in the viability of a complex product. Together with the freedom of product design, the ability to customize and the variety of products are determining factors in the competitiveness of the Supply Chain [18]. The environmental impact, health, and safety seems to be contemplated in this technology that marks a trend in terms of resource consumption [16,18]. The impact of the technology on production strategy, technical requirements and distribution is still to be resolved [18].

Within the study that allows the development of a conceptual model of the Supply Chain using Blockchain technology, it becomes evident that, as in companies in the aerospace sector, the top management is responsible for making strategic decisions and therefore for designing and implementing sustainability in the organization [15,19].

### 3.2. Shipbuilding

In the case of shipbuilding, there are only five articles that meet the search criteria from which only one had to be removed, which will be analyzed below. In the same way as in the previous section covering the aerospace sector, this section also considers the Engineer-to-order type of production. Thus, this sector faces the same problem of susceptibility to external breakdowns due to the difficulty of small enterprises to adapt to Industry 4.0 [10]. In the same way, the Lean methodology provides benefits in the Engineer-to-order environment, together with the relationship between digital and information technology of the I4.0, which is considered as an established term in the Lean Supply Chain [20].

In addition to the Lean paradigm, there are other Supply Chain Paradigms studied for the shipbuilding sector such as Green, Agile, and Resilient, which in combination with the enabling technologies stand out from the others in Big Data Analysis focused on the reduction of emissions. Data processing enables the reliability and security of data to quantify CO_2_ emissions from ships and provide information on energy efficiency parameters [21]. Other techniques include optimizing the energy efficiency of the ship by analyzing the energy transfer between the hull, propeller, and main engine; analyzing the optimal engine speeds [22]. After analyzing the data collected, including sea currents, waves, and winds, along with engine logging data, location, and speed, it is possible to predict ship performance, reduce fuel consumption, and thus reduce emissions [23], even by analyzing historical data as a basis for estimating future accidents [24]. 

Other technologies focused on Supply Chain sustainability are Cloud Computing, Cybersecurity, and Blockchain [25]. Cloud Computing studies the optimization of virtual machine placement. This is a great challenge in terms of the number of physical machines with the aim of reducing energy costs and waste of resources, in addition to minimizing operating expenses dedicated to the target platform [26], in collaboration with other technologies allowing a rapid diagnosis of system efficiency, in particular engine breakdown [27], in addition to collaborating in the sustainable development of the marine economy [28]. 

Cybersecurity has an important role to ensure the safe operation of ships, in addition to improving the environmental safety of the oceans. With the intention of complying with international regulations, the available resources are studied by analyzing the methods and policies of maritime cybersecurity that guarantee these aspects [29]. At the same time, there are publications that aim to inform staff to help protect cyberspace from adversaries through an introductory view of systems that help manage cyberspace security that simplifies the complexity of cyberspace and the variety of possible attacks.

As for the energy efficiency of cryptocurrencies, Blockchain technology tries to implement and change to more efficient algorithms such as the Proof of Stake (PoS), leaving behind the use of the Proof by Work (PoW) algorithm used to achieve energy sustainability [30].

In the aeronautical sector, these technologies have also taken on a leading role with regard to the sustainability of the Supply Chain, but there is no evidence of this from Cybersecurity. This does not mean that the sector has not focused on the study of this technology; there is evidence related to the characterization of digital manufacturing systems, identification of threats and vulnerability, control, and determination of risks [31].

There are studies that show the benefits of Blockchain, Internet of Things, and Fog Computing technologies in the application to a system that allows the identification and tracking of the pipes of a ship during its construction [32]. Likewise, no publications on Fog Computing technology have been published in the aeronautical sector as a technology that drives the Supply Chain and its sustainability, although in the same way as Cybersecurity, it does in other areas [33]. 

The Internet of Things has also been applied in other sector companies revealing a great impact on the performance of the Supply Chain and highlighting the potential for improvement not only in the economic but also in the environmental and social sustainability aspects. Its use allows a sustainable development in collaboration with the strategic and organizational management of the companies. In addition, it offers solutions attending to criteria such as the management of services or operations from the perspective of business based on intelligent operations [34].

### 3.3. Automotive

In the case of the automotive industry, there are a total of 54 publications, of which two have been eliminated and three have been evaluated in the sectors studied. In this case, and to consider the difference of the previous sectors, the type of production corresponds to the mass production; however, the Lean methodology is also present in this type of production.

One of the improvements in the operation and control of the plant is done through the relationships of the key performance indicators (KPIs). This performance measurement system of a Lean production system provides answers at the strategic, tactical, and operational levels in the implementation of I4.0 projects [35]. In addition to the contribution of Lean guaranteeing an efficient use of resources, in combination with Agile, they act as drivers for the general improvement of performance. As a decision support tool for decision making by identifying potential I4.0 technologies, the Lean–Agile combination adopts strategies that help achieve the overall objectives of the organization [36].

Another possible combination with Lean that is used as a lever to strengthen relationships is with Green practices. The result in this case would be Green Supply Chain Management, where Lean facilitates the collaboration with suppliers and environmental programs. At the same time, following a process innovation strategy based on I4.0 technologies, in addition to improving the Lean effect, leads to better economic results. However, companies will have to choose to obtain better performance by charging suppliers in environmental programs or by investing in I4.0 technologies, but not in both [37]. This is because innovation in technologies does not have the same impact on the Green Supply Chain; if the intention is to improve performance by targeting technologies, then Green is not being improved and vice-versa.

In the same way, it has been demonstrated that I4.0 technologies do not improve the performance of the Lean Supply Chain, and it can be negative to think that better results will be achieved by acquiring a technology than through management practices [38]. However, there are other studies that indicate that the Green and Lean approach can improve the content of I4.0 by adapting product and process design, manufacturing planning and control, cooperation with suppliers, shared information and customer energy and value through flexibility and process re-engineering, with communication between Supply Chain players being essential. All this makes the Supply Chain more flexible and visible and can be made possible through I.40 enabling technologies [12].

Supported by these information and communication technologies and Lean Manufacturing management methods, a new generation of manufacturing systems is born, which is called a Small Scale Intelligent Manufacturing System that is capable of generating value and meeting customer demands. In addition, in order to carry out Green Manufacturing, a Closed-loop Supply Chain model was developed [39]. This concept of Closed-Loop is not new; it was introduced by Solvang in 2007, defining it as a Supply Chain without waste [40], and it is related to a more current concept such as the circular economy.

This circular economy is favored by the interconnectivity promoted by Industry 4.0 allowing for real-time data collection, communication, and data analysis [41], although the transition between Industry 3.0 and 4.0 presents barriers between the Circular Supply Chain and Industry 4.0 [42]. Among the barriers to implementation of I4.0 are the workforce capable of understanding Industry 4.0, ineffective legislation and control, and short-term corporate objectives. These barriers, combined with the lack of funding for I4.0 initiatives, are causing organizations to develop an integrated strategic approach that is capable of utilizing the improved knowledge of I4.0 and the circular economy in order to take advantage of the increased profits from products and process designs that promote energy efficiency [43].

To achieve the effectiveness of Industry 4.0 in the sustainability of the Supply Chain, initiatives are identified from the organizational, legal, and ethical perspective and technological strategies. Within these technological strategies are the need for integration of technological platforms, data-sharing protocols, and a lack of internet-based network infrastructure [44]. Data-based technology and operations provide opportunities for new methods and operations to become an adopter of Industry 4.0 [45].

**Figure 4:**
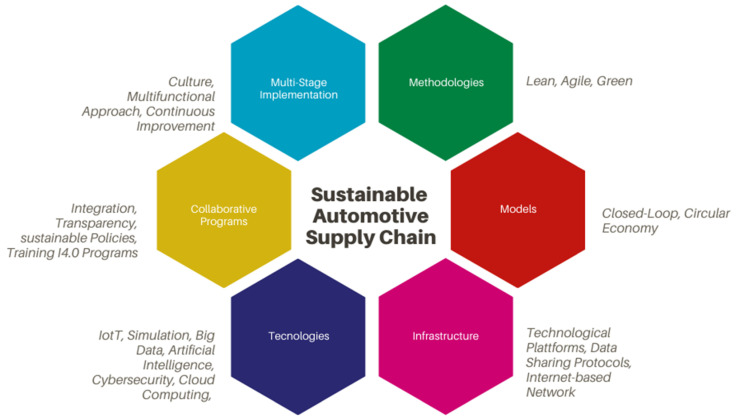
Sustainable Supply Chain facilitators.

In order to know the facilitators of the sustainable Supply Chain, Figure 4 shows the most significant ones looking for the highest demand for digital, horizontal, and vertical integration and End-to-End.

The framework of Supply Chain processes in which the Internet of Things becomes highly important had already been appreciated earlier in the aerospace sector [14]. And the impact it has on the performance of the Supply Chain by improving economic, environmental and social sustainability aspects in shipbuilding [14]. It could therefore be said that the Internet of Things and environmentally friendly practices are the most influential factors in becoming a sustainable and industry-compliant organization 4.0 [46]. 

This is not the case with Additive Manufacturing, despite the fact that its adoption has many effects from the viability of a complex product, the freedom of design or the ability to mass customize, there are still contradictions with regard to the complexity and flexibility of the Supply Chain in addition to not being profitable in the automotive industry [18]. Just the opposite of the other two sectors.

Furthermore, there are studies that show how simulation boosts the flexibility and efficiency of the automotive Supply Chain by using simulation based on multi-objective optimization and developing a decision support model [47]. This flexible simulation-based approach allows risks to be assessed prior to implementation with a positive impact on Supply Chain risk management, saving many real resources, which makes the Supply Chain more sustainable [48,49]. 

Another way to achieve sustainability in production is through the use of Just in Time material in the assembly lines; this is achieved by implementing decentralized logistics areas known as supermarkets. At the same time, it was observed how the cost of shipping material across the assembly line is the most influential factor in reducing the total cost of the supermarket. It was through simulation that the optimum location of these supermarkets on the assembly lines was optimized [50]. Hence, the simulation allows us to optimize from a particular point of view any necessary movement by making iterations until the optimal solution is reached. This same concept is used in previous sectors, but there are no simulation-related applications for it.

The simulation also served as a semantic validator of Big Data, due to the fact that the Big Data technology showed indetermination when analyzing the data that could be solved through simulation. This shows that Big Data technology requires improvement [51]. However, it is the analysis of Big Data that drives artificial intelligence to achieve sustainable manufacturing and circular economy capabilities [52].

The expected connection in the automotive factories make the amount of shared data very large through the activities of the Supply Chain and in the interaction of product and service in the cloud. This shows the need to implement Cybersecurity through the integration of Supply Chain management—marketing integration [53]. In addition to marketing integration, the other areas addressed within the Supply Chain also benefit from Cybersecurity.

An adaptation of cloud computing with the use of robots, cloud robotics, are key to the virtual creation and integration of computational and physical processes resulting in the Cyber–Physical–Systems key to the transition to the sustainable digital world [54]. These systems make it necessary to analyze Cybersecurity risks in a globalized Supply Chain. Some occur due to cyber-attacks that cause an operational disruption in the SC; others cause an operational disruption affecting the entire Supply Chain, and others are produced by an inappropriate interaction between man and machine [55]. In addition to Cybersecurity, security in the traceability of operations is also necessary, for which a reference architecture of the applicability of Blockchain technology is necessary as well [56].

However, it seems that most companies prefer the implementation of only one technology to the adoption and integration of several. Most of them invest in the Internet of Things, Cloud Computing, or Radio Frequency Identification due to the optimization of resources, ease of access from anywhere, or for decision-making based on visibility. Others choose Big Data Analytics because of the speed in detecting failures with a better customer service and reduction of preventive maintenance. Furthermore, some companies rely on Blockchain to improve the traceability and transparency, which increases trust with stakeholders [57]. Several of these technologies such as Robotics, Automated Guided Vehicles, or Additive Manufacturing help reduce wasted resources and emissions by setting up a collaborative program. Thereby, when innovation costs are shared, the motivation to invest more is greater, and this translates into better Supply Chain performance [58]. It can be said that either the actors in the Supply Chain work collaboratively and support each other, or there will be no success in the performance of the Supply Chain [38]. It seems fundamental for the growth of Industry 4.0 and the coordination between the entities of the Supply Chain to establish models in daily environments, competition, and cost-sharing contracts [59].

In spite of seeking solutions such as collaboration, there is a lot of resistance that companies encounter when it comes to putting into practice the management changes that a sustainable Supply Chain carries out. As mentioned above, the size of companies has an influence, making it easier for larger companies to implement changes than for smaller ones. Another barrier is found at the level of employees and middle management in the face of increased control and performance measurement in real time, fearing changes in management [60,61] in addition to the lack of knowledge on the part of the managers of knowing if they will return the investment and will obtain benefit nor in time [62]. It could be said that one of the biggest problems the automotive industry faces is in management and organization [63]. There are also barriers due to lack of knowledge of I4.0 by suppliers [61]. The lack of technological infrastructure also makes implementation more difficult considering that there is no management support for the implementation of I4.0 [61,62].

On the other hand, there are findings that show that neither customer loyalty nor satisfaction is relevant to the success of Supply Chain management. The customer experience will be a differentiator in the future, and it will work to maintain the support of the rest of the factors [64].

However, there are still areas to be exploited that can be beneficial in the automotive industry [65]. In order to help the leaders of the companies make their plants intelligent, it is clear that there is a need for integration, collaboration, and transparency of all the members of the chain [66]. Leaders are encouraged to establish sustainable policies, training programs focused on I4.0 and to consider I4.0 as a strategic decision to improve costs, reduce resources and energy consumption, and contribute to the development of healthy societies [44]. However, this integration, behavior, and trust will be reflected when it is manifested by including the concept in the vision and mission of their organizations [13].

One of the proposals still to be developed is the servicing of Supply Chain management with respect to I4.0 applications [67]. Another is the implementation of I4.0 concepts at multiple levels of the Supply Chain. Within this multi-stage implementation proposal, they discourage talkers that go from a cultural, multifunctional approach and continuous improvement. It proposes to start from the focus organization for later integration of the partner organizations until arriving at the intelligent factory where the Supply Chains are connected among themselves and with their systems and the machines are linked to a common network system [68]. Finally, the proposal relating to installations and the application to the recovery of the value of the product at the end of its life cycle could be mentioned [69].

**Figure 5:**
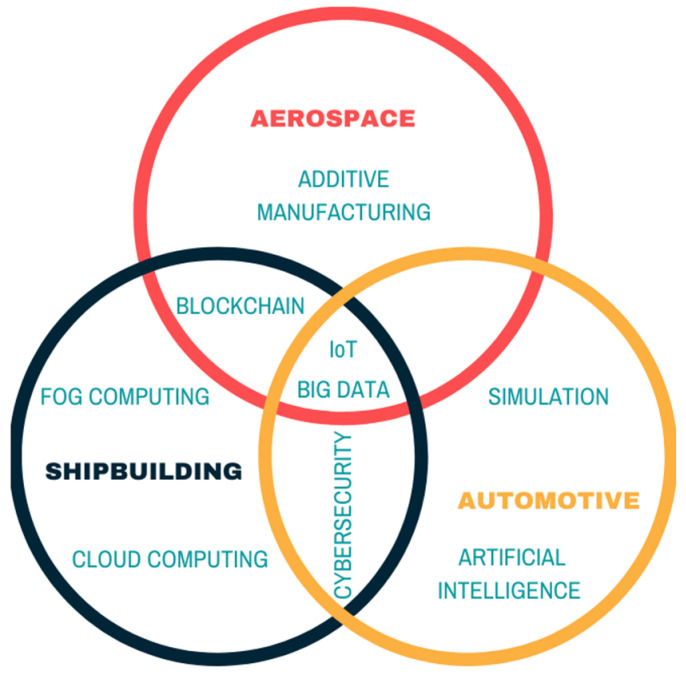
Focus technologies in Supply Chain Management (SCM) digitalization.

**Figure 6:**
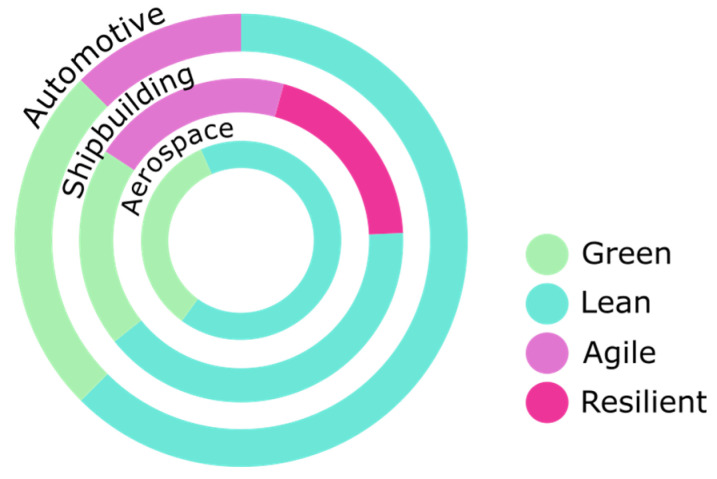
Sustainable Supply Chain-driving methodologies.

Figure 5 shows, as a summary, the technologies that each of the sectors studied considers applicable to boost sustainability in the Supply Chain. It shows how only Big Data and the Internet of Things are common to all three sectors. Similarly, Figure 6 shows the methodologies and practices that each of the sectors studied apply to the sustainable Supply Chain.

### 3.4. Key Points Overview

In this last section, a summary of the most significant aspects of each sector studied is included, as shown in Table 1, in order to establish a comparison between them. The facilitators referred to in Figure 4 for the automotive industry supply chain are taken as a reference.

## 4. Conclusions

In the aerospace sector, there is a tendency among companies that are committed to a sustainable 4.0 Supply Chain to be concerned that the breakdown will come from small external companies in the Supply Chain. Even so, they adopt Lean and Green methodologies considering their impact on performance. 

With regard to the macro processes described in the introduction, the aerospace sector is committed to managing them through the Internet of Things applications, improving both the relations between the participants and with regard to the adoption of sustainable actions. In addition, there is evidence of the use of other technologies such as Big Data, Additive Manufacturing, and Blockchain, which are also focused on the implementation of sustainability in the Supply Chain.

Similarly, in the shipbuilding sector, there is also evidence of the concern about the ruptures caused by the smaller companies that make up the sector. In this case, the paradigms studied for this sector coincide with the aerospace sector, and the Agile and Resilient paradigms are added as well. 

There is little evidence of the implementation of different technologies in this sector, although the Internet of Things seems to be the most remarkable.

In the case of the automotive industry and changing from production to mass production, they coincide with the Lean, Green, and Agile paradigms, although there is controversy in particular regarding Lean Supply Chain and 4.0 technologies where management practices are preferable. With regard to technologies, it could be said that this sector is one of the ones that has most implemented its applications in most of them, highlighting on the one hand the additive manufacturing as, despite the advantages it has, it does not seem to give benefits in this sector. On the other hand, Simulation stands out as providing flexibility and efficiency to the automotive Supply Chain and as a facilitator together with other technologies.

It seems that the sector is committed to the implementation of the technologies in a collaborative manner among the participants in the chain and also in the implementation by stages. Furthermore, the sector has identified the barriers that prevent it from successfully implementing technologies that make the Supply Chain sustainable, and it mainly identifies the human factor in this.

Despite comparing sectors with different production systems, it can be seen how all three rely on Lean practices as necessary to make the Supply Chain sustainable. Even the automotive sector, being the one that presents more publications, prefers Lean management practices to the benefits that Industry 4.0 technologies could bring. It could be said that Lean practices should be intrinsic to the company and that any technology to be implemented should not displace these practices.

With regard to technologies, all three sectors reveal a strong interest in the Internet of Things as being paramount for the sustainability of the Supply Chain. At the same time, Big Data and Blockchain are two technologies that also demonstrate contributions to sustainability and therefore focus on all three sectors. However, additive manufacturing is appropriate for the aerospace and shipbuilding sector, while the automotive sector does not find the full benefit. The technology that this sector is interesting in is Simulation, contributing considerably directly to the Supply Chain and indirectly as support to other technologies.

Finally, the contribution of the automotive sector to collaborative approaches to change management to smart factories should be highlighted, which at the same time would help alleviate the concern of the aerospace and shipbuilding sectors about the source of external breakdowns of components in the Supply Chain.

## Figures and Tables

**Table 1:** Comparison of SC facilitators.

	Aerospace	Shipbuilding	Automotive
Methodologies	Lean practices improve SC performance [11,12,70,71,72,73,74,75,76,77]	Lean strengthens the probability of success of Supply Chain Management [78,79]	Lean provides competitive advantages, quality, and flexibility performance [80,81] and improves dealer service through an inventory management model [82]
	Agile practices evaluate new event with restructuring suggestions [75,83,84,85]	The Agile methodology identifies improvements in the relationship between the shipyard and its suppliers [85,86]	Agile provides competitive advantages, quality, and flexibility performance [80,87] and is used as a strategy for supplier selection [88]
	Green practices make an important contribution to SC sustainability and suppliers [73,75,86,89,90,91]	Green practices contribute to a sense of social responsibility and competitive advantage [86,92]	Green practices improve the relationship between companies and green suppliers, improves the capacity to develop green products, and increases the competitiveness of companies in the market [93] and minimizes the total cost [94]
	Resilient initiatives improve SC sustainability and social improvements in safety and environmental health [70,72,73,83,95]	The resilient paradigm is compromised by the social and functional aspects of the I4.0 performance model [96]	Resilient methodologies to SC are preferable to focus on minimizing costs [97], improve the selection of sustainable and appropriate suppliers, and maximize value by developing close and long-term relationships [98]
Models	Closed-Loop SC models help increase profits by transforming and remanufacturing waste [86]	Closed-Loop SC models help increase profits by transforming and remanufacturing waste [86]	Adding value to remanufacturing practices [99] collaborating with environmental management [100]
	No evidence of Circular Economy	Circular economy helps to reduce CO_2_ emissions [101]	Circular economy provides priority solution measures to formulate effective strategies to overcome failures in the adoption of SC management [63]
	Environmental sustainability by applying the product life cycle management system [90,102,103]	Product lifecycle management (PLM) contributes to efficient control and distribution, minimizes costs, and reduces lead times [104]	Product life-cycle management (PLM) approach supports decision making [105], reduces the time to market, and satisfies the end customer needs [106]
Infrastructure	Use of technological platforms to improve logistics capacity [107] and to develop the reference architecture and define the standards to exchange electronic information securely [108]	The use of technological platforms achieves an important integration and collaboration with its suppliers and customers [109]	Through a platform with several simulation components, the control of the manufacturing systems is established [110]. In addition, an integrated platform based on a cyber–physical system provides optimal use of manufacturing resources in dynamic, real-time environments to increase efficiency and responsiveness to uncertain market changes [111]
	Data Sharing Package allows the reduction of SC inefficiencies [112]	Lightweight data format for the visualization of 3D product information and the collaboration of all SC agents in all phases of the ship lifecycle [113]	Data-sharing protocols based on Blockchain technology provide reliability [114]. Data sharing on production planning and scheduling using IoT can reduce product preparation and delivery time [115]
	Analysis, design, and performance improvement of the SC by applying the SC Operations Reference Model (SCOR) using the internet [116,117]	Web-based software framework that enables electronic collaboration between companies working together for ship repair [118,119], An open communication infrastructure guarantees the success of SC [120]	Providing benefits to remanufacturing practices through the use of Big Data using the Internet [99]
Technologies	IoT: Registering and verifying the identity of the machines simplifying the management of the assets within the connected SC [121]	IoT: Identification and tracking of the pipes of a ship during its construction [32], offering solutions for the management of services and operations [34]	IoT: Allowing connectivity for later analysis through simulation [110]. This exchange of data on production planning and scheduling using IoT can reduce product preparation and delivery time [115,122]
	Simulation: To accurately model or predict the effects of joining and fixing parts [123], analyzing SC performance [124], for decision support [125]	Simulation: Management tool [107], to solve complicated problems of SC management [126], identify the critical control point to mitigate the effects caused by the disproportion in the logistic flow [127]	Simulation: As a training tool for ship design processes [128] and as a tool for decision making [105]
	Big Data Analytics: Support for dynamic production capacity and decision making of the SC [129]	Big Data Analytics: Used to optimize the design of a vessel and to maximize efficiency and safety in an existing one [130], focused on the reduction of emissions [21,22]	Big Data Analytics: Providing advantages to remanufacturing practices [99]
	Artificial Intelligence: Adaptive resource management based on multi-agent technology [131], to produce more affordable parts, faster, and with less weight [132]	Artificial Intelligence: Using control architecture and programming of the production plant [133], focused on reducing CO_2_ [23]	Artificial Intelligence: new dimension of the relationship between financing and production [134]. Solves problems in the management of the SC that can track, communicate, analyze, and ensure the overall sustainability of the system [135]. Facilitate the execution of mechanism design-based negotiations [136]
	Cybersecurity: To derive the behavior of programs with hidden malicious operations and supporting workforce productivity [137], providing operational certainty of SC systems [138]	Cybersecurity: Improving economic, energy, and environmental aspects [96]	Cybersecurity: Threat deterrence and mitigation function [139]. Provides mechanisms for identifying generic and manufacturing-specific vulnerabilities [140]
	Cloud Computing: Providing unlimited processing to SC management [141]	Cloud Computing: Improving economic, energy, and environmental aspects [96]	Cloud Computing: Allows the collection, supply, and analysis of relevant data in all companies that make up the SC [122,142]
–	Additive Manufacturing: Supporting sustainability in CS through material recycling [143], remanufacturing of high-value parts on the reverse logistics supply chain [144]	Additive Manufacturing: Enabling design flexibility, reducing waste, and integrating subassemblies [145], Negative aspect: increased delivery time, shipping cost, inventory requirements, and transportation vulnerability [146]	Additive Manufacturing: Used during the supply stage; it changes complex subsets into a single integrated structure [147]
	Blockchain: Ensuring traceability by certified agents in the SC [148,149]	Blockchain: strengthening production security in the collaborative development process, improving the integrity and traceability of Supply Chain data [150]	Blockchain: provides reliability in the creation of protocols to share processes, business logic, and financial ledgers [114]. Guarantees the security, transparency, and visibility of the network from the origin of the SC, the reengineering of the business processes to the improvement of the security [151]
Collaborative Programs	Use of system of systems to address multi-system integration problems associated with SC [152], Collaborative Aerospace Life Cycle Systems Program that integrates from the beginning of the aerospace design process [153]	Information systems for project management with integrated approach [154], high integration and collaboration between design, manufacturing, and management functions [109]	Logistics integration through collaborative supply chain innovation [155]
	Gaining transparency between the central company and its suppliers, exchanging high-quality information leads to significant improvements in overall SC performance [156]	Through transparency, collaborative risk management in SC management shows collaborative control mechanisms [157]	Through the Blockchain technology, the security, transparency, and visibility of the network is guaranteed [151]. Focal companies increase multi-tier SC management transparency for sustainability [158]
	Through the implementation of sustainable policies with long-term strategies among the agents involved in SC [159]	Through carbon policies based on the sustainability characteristics of the region, the level of design of Supply Chain networks is improved, cost is reduced, and the environmental impact is improved [160]	The application of Green strategies to the management of CS helps companies establish innovative and effective policies [161]. Closed-Loop SC provides recommendations for sustainable policies [100]
	No evidence of the I4.0 training programs despite potential benefits to SC management [162]	No evidence of I4.0 training programs despite potential benefits to SC management [162]	Design of training tools for ship design processes through the use of simulation [128]
Multi-Stage Implementation	No evidence of culture in the sector in relation to SC	No evidence of culture in the sector in relation to SC	Implementing Green practices in the management of SC collaborates in the implementation of socio-cultural responsibility [163]
	No evidence of multifunctional approach in the sector in relation to SC	No evidence of multifunctional approach in the sector in relation to SC	Multifunctional approach using Closed-Loop SC [164]
	Continuous improvement of the quality of products and processes [165] system to define a Lean workflow [166]	Through collaborative tools that allow completely managing the SC in continuous improvement [154]	Continuous improvement to reduce stocks [167], evaluating the performance of the downstream supply chain [168,169]
